# The CO_2_ fertilization effect on leaf photosynthesis of maize (*Zea mays* L.) depends on growth temperatures with changes in leaf anatomy and soluble sugars

**DOI:** 10.3389/fpls.2022.890928

**Published:** 2022-08-19

**Authors:** Liang Liu, Lihua Hao, Yunxin Zhang, Haoran Zhou, Baoguo Ma, Yao Cheng, Yinshuai Tian, Zhijie Chang, Yunpu Zheng

**Affiliations:** ^1^School of Water Conservancy and Hydropower, Hebei University of Engineering, Handan, China; ^2^Department of Ecology and Evolutionary Biology, Yale University, New Haven, CT, United States; ^3^School of Landscape and Ecological Engineering, Hebei University of Engineering, Handan, China

**Keywords:** photosynthesis, water use efficiency, stomatal traits, leaf anatomy, total soluble sugar

## Abstract

Understanding the potential mechanisms and processes of leaf photosynthesis in response to elevated CO_2_ concentration ([CO_2_]) and temperature is critical for estimating the impacts of climatic change on the growth and yield in crops such as maize (*Zea mays* L.), which is a widely cultivated C_4_ crop all over the world. We examined the combined effect of elevated [CO_2_] and temperature on plant growth, leaf photosynthesis, stomatal traits, and biochemical compositions of maize with six environmental growth chambers controlling two CO_2_ levels (400 and 800 μmol mol^−1^) and three temperature regimes (25/19°C, 31/25°C, and 37/31°C). We found that leaf photosynthesis was significantly enhanced by increasing growth temperature from 25/19°C to 31/25°C independent of [CO_2_]. However, leaf photosynthesis drastically declined when the growth temperature was continually increased to 37/31°C at both ambient CO_2_ concentration (400 μmol mol^−1^, *a*[CO_2_]) and elevated CO_2_ concentration (800 μmol mol^−1^, *e*[CO_2_]). Meanwhile, we also found strong CO_2_ fertilization effect on maize plants grown at the highest temperature (37/31°C), as evidenced by the higher leaf photosynthesis at *e*[CO_2_] than that at *a*[CO_2_], although leaf photosynthesis was similar between *a*[CO_2_] and *e*[CO_2_] under the other two temperature regimes of 25/19°C and 31/25°C. Furthermore, we also found that *e*[CO_2_] resulted in an increase in leaf soluble sugar, which was positively related with leaf photosynthesis under the high temperature regime of 37/31°C (*R*^2^ = 0.77). In addition, our results showed that *e*[CO_2_] substantially decreased leaf transpiration rates of maize plants, which might be partially attributed to the reduced stomatal openness as demonstrated by the declined stomatal width and stomatal area. These results suggest that the CO_2_ fertilization effect on plant growth and leaf photosynthesis of maize depends on growth temperatures through changing stomatal traits, leaf anatomy, and soluble sugar contents.

## Introduction

Global atmospheric CO_2_ concentration ([CO_2_]) is projected to be between 421 and 946 μmol mol^−1^ by the end of 21st century depending on continued emission scenarios (IPCC, 2021). Meanwhile, this elevated [CO_2_] may lead to climate warming through the greenhouse effect, and thus the global surface temperature is expected to be increased by 1.5°C–5.3°C (IPCC, 2021), and the frequency of extreme high temperature events are also anticipated to increase in the near future (Barlow et al., 2015; Jin et al., 2017). Therefore, the elevated [CO_2_] and temperature may result in drastic impacts on the structure and function of the terrestrial ecosystem (Ito, 2010). For example, several studies have demonstrated that the changes in [CO_2_] and temperature generally lead to impacts on crop growth and grain yield (Ainsworth and Long, 2020; Muluneh, 2020) indirectly by altering crop phenology (Piao et al., 2019) and directly by changing leaf photosynthesis and respiration (Dusenge et al., 2019; Poorter et al., 2021), stomatal morphology and distribution (Hao et al., 2019), leaf anatomy and morphology as well as nonstructural carbohydrates and nitrogen (Xu et al., 2012).

It is well known that temperature is a common environmental factor regulating plant growth and crop yield through various physiological and biochemical processes such as leaf photosynthesis and respiration (Zheng et al., 2013, 2018; Zinyengere et al., 2013; Perdomo et al., 2017; Dusenge et al., 2019). Plant responses to temperature are fundamentally mediated by leaf photosynthesis (Abebe et al., 2016), which may be upregulated or downregulated depending on whether the temperature is below or above what is optimal for plants (Jin et al., 2011). Elevated temperature can stimulate leaf photosynthesis by enhancing the carboxylation efficiency (Contran et al., 2012) through increasing ribulose-1,5-bisphosphate carboxylase/oxygenase (Rubisco) concentration and activity (Mueller-Cajar et al., 2014) within a certain temperature range (Jin et al., 2011). However, temperatures above optimum may result in down-regulation of leaf photosynthesis by disrupting the structure of chloroplasts (Hao et al., 2019), damaging the function of photosystem II (Janka et al., 2015), and suppressing the activation state of Rubisco (Hao et al., 2019). In addition to elevated temperature, *e*[CO_2_] may also lead to profound impacts on plant growth and crop yield (Abebe et al., 2016). Most previous studies have found that the leaf photosynthesis and growth of C_3_ plants are drastically enhanced under elevated [CO_2_] (Yu et al., 2012; Zhang et al., 2020) through the “CO_2_ fertilization effect,” because the Rubisco of C_3_ plants is not CO_2_-saturated at the current atmospheric [CO_2_] (Yu et al., 2012). However, no consistent conclusions have been drawn on the potential mechanisms of C_4_ plants such as maize in response to enriched [CO_2_] (Kim et al., 2007; Manderscheid et al., 2014; Abebe et al., 2016; Kellner et al., 2019). Most previous studies have shown that elevated [CO_2_] barely affected the leaf photosynthesis and plant growth of C_4_ species mainly due to the anatomical specialization associated with the CO_2_ concentrating mechanism (Kim et al., 2007; Ruiz-Vera et al., 2015). However, several studies claimed that both the leaf photosynthesis and plant growth of C_4_ plants can be substantially stimulated under enhanced [CO_2_] (Driscoll et al., 2005; Abebe et al., 2016; Kellner et al., 2019). Furthermore, elevated [CO_2_] and temperature may also have combined effects on the function of C_4_ plants (Manderscheid et al., 2014; Abebe et al., 2016), and thus ecosystem functioning under future climate change (Ito, 2010). Earlier studies have demonstrated that the sensitivity of photosynthesis to CO_2_ in C_4_ plants might be enhanced by elevated temperature (Sage, 2002; Lee, 2011), even if the impacts of high temperature stress on C_4_ plants can also be partially mitigated by CO_2_ fertilization effect (Abebe et al., 2016). However, so far, the underlying physiological processes and mechanisms of the CO_2_ fertilization effect alleviating high temperature stress on C_4_ plants is still unclear. Therefore, identifying the optimal growth temperature of C_4_ plants and understanding the physiological processes and mechanisms of enriched [CO_2_] mitigating high temperature stress on plant growth of C_4_ crops are pivotal to accurately estimate the potential risks and impacts of climate change on global agriculture productivity and grain yield (Dusenge et al., 2019).

In addition to physiological processes, plants may also alter leaf structures and stomatal traits to respond to elevated CO_2_ and temperature (Smith et al., 2012; Zheng et al., 2019). Several studies have found that plants grown at higher temperatures had thinner leaves due to the reduced size of mesophyll cells (Jin et al., 2011; Hao et al., 2019). Elevated temperature can also change stomatal traits such as stomatal size, shape and density (Hao et al., 2019), which are closely related to leaf-level photosynthesis and transpiration. Moreover, elevated [CO_2_] may also have profound effects on leaf structures and stomatal traits (Fan et al., 2020), although the conclusions are generally inconsistent (Rengifo et al., 2002; Smith et al., 2012; Fan et al., 2020). Previous studies have reported that leaf thickness and/or stomatal density are usually increased (Smith et al., 2012; Fan et al., 2020) or decreased (Zheng et al., 2019) when C_3_ plants are grown at higher [CO_2_], and other studies also found that the leaf structures and/or stomatal traits of C_4_ plants were obviously affected by increased [CO_2_] (Driscoll et al., 2005; Habermann et al., 2019). These inconsistent conclusions suggested that the different responses of leaf structures and stomatal traits to elevated temperature and [CO_2_] are dependent upon plant species and/or functional groups (Smith et al., 2012; Habermann et al., 2019). Furthermore, plants’ responses to elevated temperature and [CO_2_] are not only associated with the changes in leaf physiological processes and structural traits, but also related to chemical compositions of leaves such as soluble sugars and starch (Hao et al., 2019; Zheng et al., 2019). Most previous studies have reported that elevated temperature usually reduces the content of leaf soluble sugars (Jin et al., 2011; Hao et al., 2019), but other studies have argued that elevated temperature may barely change the leaf soluble sugars (Xu et al., 2012) or even increase the nonstructural carbohydrates of plants (Zhang et al., 2017). Moreover, elevated [CO_2_] usually stimulates plant biomass accumulation and enhances grain yield of C_3_ crops due to the boosted leaf photosynthesis and increased nonstructural carbohydrates from CO_2_ fertilization effect (Zheng et al., 2019), but the effect of enriched [CO_2_] on the nonstructural carbohydrates of C_4_ plants is still unclear (Leakey et al., 2006; Faria et al., 2018). For example, Habermann et al. (2019) found that elevated [CO_2_] substantially increased the contents of starch in leaves of the C_4_ forage species *Panicum maximum*, but Leakey et al. (2006) claimed that elevated [CO_2_] had little effect on the soluble sugars and starch of maize leaves based on a Free-air CO_2_ Enrichment (FACE) experiment. These contradictions between different studies indicated that the stimulating effect of elevated [CO_2_] on nonstructural carbohydrates of plants may highly depend on the growing temperatures and species/genotypes (Song et al., 2014; Faria et al., 2018). Nevertheless, few studies examined the combined effects of elevated [CO_2_] and temperature on leaf chemical composition such as nonstructural carbohydrates, which is closely associated with the photochemical and biochemical processes of C_4_ plants such as maize (Zheng et al., 2013).

Maize (*Zea mays* L.) is one of the most important crops in many regions throughout the world, which accounts for more than 30% of global cereal production (Haden et al., 2021). Therefore, understanding the mechanisms of maize plants in response to elevated [CO_2_] and temperature is critical to projecting the potential risk of climate change on global agriculture productivity. So far, however, most studies have examined the response of C_3_ species to elevated [CO_2_] or temperature (Xu et al., 2012; Dusenge et al., 2019), while few studies have focused on C_4_ species in response to future climate change (Abebe et al., 2016) and especially on the combined effects of elevated [CO_2_] and temperature on leaf photosynthesis through changes in stomatal traits, leaf anatomy and leaf biochemistry of maize. Moreover, several modeling studies have projected that the negative impacts of climate change on global agriculture productivity can be partly mitigated or even offset by elevated [CO_2_] through the strong CO_2_ fertilization effect (Pan et al., 2014; Kassie et al., 2015), thus current climate change models may overestimate the impacts of climate change on global crop yield (Zinyengere et al., 2013). Therefore, we conducted this study with environmental growth chambers controlling two CO_2_ levels (400 and 800 μmol mol^−1^) and three temperature regimes (25/19°C, 31/25°C, and 37/31°C) to test the following hypotheses: (1) Maize has an optimal temperature for leaf photosynthesis and plant growth, which can be constrained by high temperatures above the optimum (HY1); (2) The negative effects of high temperature on leaf photosynthesis and plant growth in maize can be mitigated by elevated [CO_2_] through the CO_2_ fertilization effect (HY2); (3) This CO_2_ fertilization effect can be explained by the changes in nonstructural carbohydrates in maize leaves at high temperatures (HY3); (4) The enhanced leaf photosynthesis and water use efficiency by higher [CO_2_] is associated with the changes in stomatal traits and leaf anatomy of maize plants under high temperatures (HY4).

The objectives of the current study were: (1) to evaluate the optimal temperature for maximizing the CO_2_ fertilization effect on maize plants, (2) to examine the combined effects of elevated CO_2_ concentration and temperature on the growth of maize plants, and (3) to investigate the underlying mechanism through which growth temperature modulates CO_2_ fertilization effect on leaf photosynthesis of maize plants by modifying stomatal traits, leaf anatomy, leaf photosynthesis and leaf biochemistry.

## Materials and methods

### Growth chamber experiment

A split-plot experimental design was arranged consisting of two factors (CO_2_ and temperature) with CO_2_ concentration as the main plot (two CO_2_ levels) and temperature (three temperature regimes) as the subplot. Six environmental growth chambers (Model BDP-2000, Ningbo Prandt Instrument Co., Ltd., China) were employed for sustaining plant growth and controlling CO_2_ concentrations, where the CO_2_ concentration in three environmental growth chambers was supplied with 400 μmol mol^−1^ (*a*[CO_2_]), and the target CO_2_ concentration in the other three growth chambers was maintained at 800 μmol mol^−1^ (*e*[CO_2_]). In order to reduce air pollution on plants, these growth chambers were supplied with high purity CO_2_ source (99.99%) from a CO_2_ bottled tank. The other environmental factors in the six growth chambers were maintained similarly during the establishment of maize plants (30 days) with photosynthetic photon flux density (PPFD, 1,000 μmol m^−2^ s^−1^), growth temperature regime (25/19°C, day/night), and photoperiod (8:00–20:00). The inside air temperature of each growth chamber was maintained within 0.5°C throughout the experiment. Meanwhile, the relative air humidity was controlled at 60%–65% in each environmental growth chamber. Furthermore, these [CO_2_] values in both the *a*[CO_2_] or *e*[CO_2_] growth chambers were about 20 μmol mol^−1^ around the target [CO_2_]. In the current study, we selected a maize cultivar (*Zea mays* cv. *Zhengdan 958*) that is widely planted in China (Zheng et al., 2013). Maize seeds were grown in 50 cm height plastic pots with an area of 531 cm^2^ on top and 380 cm^2^ at bottom, and these pots were filled with local soil (36% clay, 50% silt, and 14% sand with a gravimetric bulk density of 1.58 g cm^−3^). The space inside these environmental growth chambers (1.83 m high × 1.79 m long × 0.68 m wide) was large enough for maize plant growth. Then, four pots were randomly set up in each of the environmental growth chambers, and thus the four pots in each environmental growth chamber were the biological replications (*n* = 4). Maize plants were fertilized with half-strength Hoagland’s solution (150 ml per pot) weekly during the establishment (30 days) and treatment (60 days) periods (Zheng et al., 2019). Additionally, maize plants were relocated among different growth chambers once per week during the vegetative growth of maize plants to minimize the confounding effect of environmental variation between different growth chambers.

### Measuring plant height, leaf traits, and plant biomass

Plant height, leaf length and maximum width were measured with a ruler, while leaf area was estimated by an area meter (LI-3000, Licor, Lincoln, NE, United States). Plant biomass was obtained by harvesting and de-potting the leaves, stems and roots with scissors. Then, plant tissues were placed in paper bags and oven-dried at 85°C for 72 h to a constant weight. The dry weight of maize plants was quantified with an electronic scale.

### Measuring leaf gas exchange and chlorophyll fluorescence

After 60 days of treatment, a new fully expanded leaf from each pot was randomly chosen to measure leaf gas exchange. This was repeated for each of the four pots in each of the environmental growth chambers. We employed a portable photosynthesis measurement system (Li-6400XT; Licor, Lincoln, NE, United States) to measure leaf net photosynthetic rates (*P*_n_), leaf stomatal conductance (*g*_s_), leaf transpiration rates (*E*), and leaf dark respiration rates (*R*_d_) at different growth temperatures and the PPFD of 1,000 μmol photons m^−2^ s^−1^ with a red-blue light source.

The sampled leaves were placed in dampened and dark bags for half an hour to ensure maximum re-oxidation of PS*II* reaction centers. Then, the maximum photochemical efficiency of PSII (*F*_v_/*F*_m_) was determined by a photosynthesis efficiency analyzer (Hansatech, Hansatech Instrument LTD, England) during the application of a 9,000 μmol photons m^−2^ s^−1^ flash for 0.8 s.

### Measuring stomatal traits

Four fully expanded leaves were randomly selected from four different maize plants in each of the chambers and stomatal characteristics were determined as described by Xu (2015). Firstly, colorless nail polish was spread onto the tip, middle, and base sections on both the adaxial and abaxial leaf surfaces. Then, the dried nail polish molds were peeled off after 30 min, mounted onto microslides and covered with micro-covers. Then, the microslides were viewed and photographed under a microscope (DM2500, Leica, Wetzlar, Germany) equipped with a digital camera (DFC 300-FX, Leica, Wetzlar, Germany). We randomly selected one image from each leaf section (tip, middle, and base section) on the two surfaces (adaxial and abaxial surfaces) of each leaf (1 image × 3 sections × 2 surfaces = 6 images). We also randomly selected two stomata from each image to measure the stomatal aperture length, width and area using the AutoDesk program on the adaxial surface and abaxial surface, respectively. Furthermore, stomata at each leaf section of two surfaces were counted and averaged to calculate stomatal density (No. mm^−2^) and stomatal area index (μm^2^ mm^−2^).

Electronic microphotographs of stomata on the abaxial surface were also obtained using a scanning electron microscopy (XL30-FEG, FEI, Eindhoven, Netherlands). Specifically, one fully expanded leaf was selected from each maize plant and then one leaf piece (2 mm × 2 mm) was snapped from the middle section of the selected leaves. The leaf piece was fixed with a fixative solution consisting of 2.5% (v/v) glutaraldehyde (0.1 M phosphate buffer, pH 7.0). Then, the samples were critical point dried, mounted on stubs, and coated with gold in a high-vacuum evaporation unit.

### Measuring leaf anatomy

The internal anatomies of maize leaves exposed to different temperatures and CO_2_ concentrations were examined using leaf cross sections under a light microscope (DM2500, Leica, Wetzlar, Germany) as described by Sage and Williams (1995). Fully expanded maize leaves were sampled and then dehydrated in a series of increasing concentrations of alcohol. These prepared leaf samples were embedded in paraffin and transversely sectioned with a microtome (Leica RM2245, Heerbrugg, Switzerland) in the laboratory. Leaf cross sections were snapped from the middle section of maize leaves and images of leaf cross sections were used to analyze leaf anatomical features with AutoCAD. Then, two cross-sectional images per leaf were selected to measure the anatomical parameters. We measured the leaf thickness at two points per cross section, and the interveinal distance between two adjacent vascular bundles per cross section (Pengelly et al., 2010). Vascular bundle tissue area and bundle sheath tissue area were also measured with AutoCAD software. Bundle sheath index was defined as the bundle sheath tissue area per cross-sectional area, calculated as bundle sheath tissue area/(interveinal distance × leaf thickness) × 100%. Vascular bundle index was defined as the vascular bundle tissue area per leaf cross-sectional area, calculated as vascular bundle tissue area/(interveinal distance × leaf thickness) × 100%.

### Analyzing leaf biochemical compositions

Maize leaves were sampled and then oven-dried at 75°C for 48 h. Leaf samples were ground to fine powder with a ball mill (MM2, Fa. Retsch, Haan, Germany). Nonstructural carbohydrates of leaf samples were assayed according to Way and Sage (2008). Glucose, fructose, sucrose, and starch concentrations were measured by spectrophotometrical methods (UV-1750, Shimadzu Corp., Tokyo, Japan) using a glucose assay kit (GAHK-20, Sigma, St Louis, MO, United States).

### Statistical analysis

We used a split-plot design two-way ANOVA to test the main and interactive effects of CO_2_ concentration and temperature on the plant growth, stomatal morphology, leaf anatomy and nonstructural carbohydrates of maize. Then, for the anatomical characteristics, stomatal traits, physiological and biochemical variables on which CO_2_ concentration or temperature showed significant effects, we employed the pairwise comparison method of Student–Newman–Keuls to compare the statistically significant differences among the treatments at *p* < 0.05 level using the SPSS 13.0 software (SPSS Inc., Chicago, IL, United States). We also employed linear regressions for estimating the relationships among leaf transpiration rates, stomatal traits, and leaf characteristics as well as the relationships between *P*_n_ and soluble sugars.

## Results

### Effects of CO_2_ concentration and temperature on the growth of maize plants

**Figure 1:**
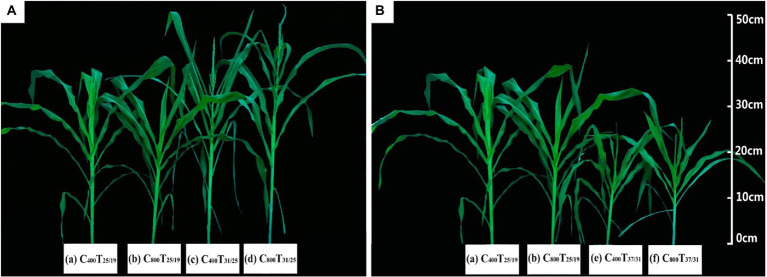
The appearance of maize grown at two CO_2_ concentrations under three temperature regimes. Note that the maize plants under 31/25°C were taller and bigger than those plants grown at 25/19°C independent of CO_2_ concentrations **(A)**. By contrast, the maize plants under 37/31°C were shorter and smaller than those plants grown at 25/19°C **(B)**. The (a-d) in figure indicates different treatments of growth temperature and CO_2_.

The total biomass and leaf biomass were increased by 10.9% and 15.7% with increasing CO_2_ concentration from *a*[CO_2_] to *e*[CO_2_] under the highest temperature regime (37/31°C), indicating elevated [CO_2_] boosted the plant growth of maize under the highest temperature regime (Figure 1; Table 1). However, this CO_2_ fertilization effect on the plant biomass of maize plants drastically diminished under lower growth temperature regimes (25/19°C and 31/25°C), as evidenced by the non-significant difference in plant biomass under differing CO_2_ regimes. In addition, we found a very strong temperature effect on the biomass accumulation of maize plants, especially the high temperature regime (37/31°C) significantly decreased the total biomass by 53.2% under *a*[CO_2_] and 46.6% under *e*[CO_2_], compared with the lower temperature (25/19°C). Interestingly, we also found that growth temperature changed the phenology of maize plants as evidenced by the longer silking stage when maize plants were subjected to the high temperature regime of 37/31°C (Table 1). In addition, an interactive effect of [CO_2_] and temperature on plant growth of maize was not detected in this study (Table 1).

**Table 1:** Effects of elevated [CO_2_] and temperature on the growth of maize plants.

[CO_2_] (μ mol mol^−1^)	Temperature (°C)	Biomass (g plant^−1^)				Plant height (cm)	Leaf area (cm^2^ plant^−1^)	Leaf length (cm)	Leaf width (cm)	Days to silking (days)
		Total biomass	Root	Stem	Leaf					
	25/19	40.6 (1.7)^a^	8.3 (1.0)^a^	16.3 (1.9)^a^	16.0 (1.9)^a^	42.6 (1.7)^b^	3,705 (189)^a^	93.3 (2.6)^a^	7.3 (0.9)^a^	47.5 (0.6)^b^
400	31/25	34.4 (1.3)^b^	7.5 (0.9)^a^	14.7 (1.6)^a^	12.2 (0.8)^b^	53.5 (1.6)^a^	2,980 (122)^b^	88.8 (4.6)^ab^	6.5 (0.8)^a^	41.0 (1.8)^c^
	37/31	19.2 (1.3)^c^	3.9 (0.8)^b^	6.6 (1.0)^b^	8.9 (0.6)^d^	29.8 (1.5)^c^	1855 (89)^d^	70.0 (2.9)^c^	4.0 (0.3)^b^	53.3 (1.0)^a^
	25/19	40.7 (1.8)^a^	7.9 (0.6)^a^	16.7 (1.6)^a^	16.1 (1.7)^a^	43.9 (0.6)^b^	3,725 (135)^a^	93.3 (1.7)^a^	7.1 (0.9)^a^	47.0 (0.8)^b^
800	31/25	36.0 (2.5)^b^	8.2 (0.5)^a^	15.0 (1.6)^a^	12.8 (0.7)^b^	54.8 (1.1)^a^	2,988 (91)^b^	86.8 (3.7)^b^	6.1 (1.0)^a^	40.3 (1.0)^c^
	37/31	21.3 (1.5)^c^	4.2 (0.3)^b^	7.0 (0.8)^b^	10.3 (0.5)^c^	30.8 (1.3)^c^	2023 (109)^c^	71.0 (2.9)^c^	4.4 (0.5)^b^	52.5 (1.3)^a^
[CO_2_]		0.09	0.50	0.54	0.09	0.12	0.11	0.80	0.96	0.08
Temperature	*p*-values	0.001	0.001	0.001	0.001	0.001	0.001	0.001	0.001	0.001
*C* × *T*		0.29	0.42	0.98	0.31	0.90	0.20	0.64	0.65	0.43

Values given are the means (standard deviation) for plant biomass (*n* = 4), plant height (*n* = 4), and leaf area (*n* = 4), and the values were compared with Student–Newman–Keuls. Different lowercase letters indicated *p* < 0.05 and the same lowercase letters indicated *p* > 0.05.

### Effects of CO_2_ concentration and temperature on leaf gas exchange and chlorophyll fluorescence

**Figure 2:**
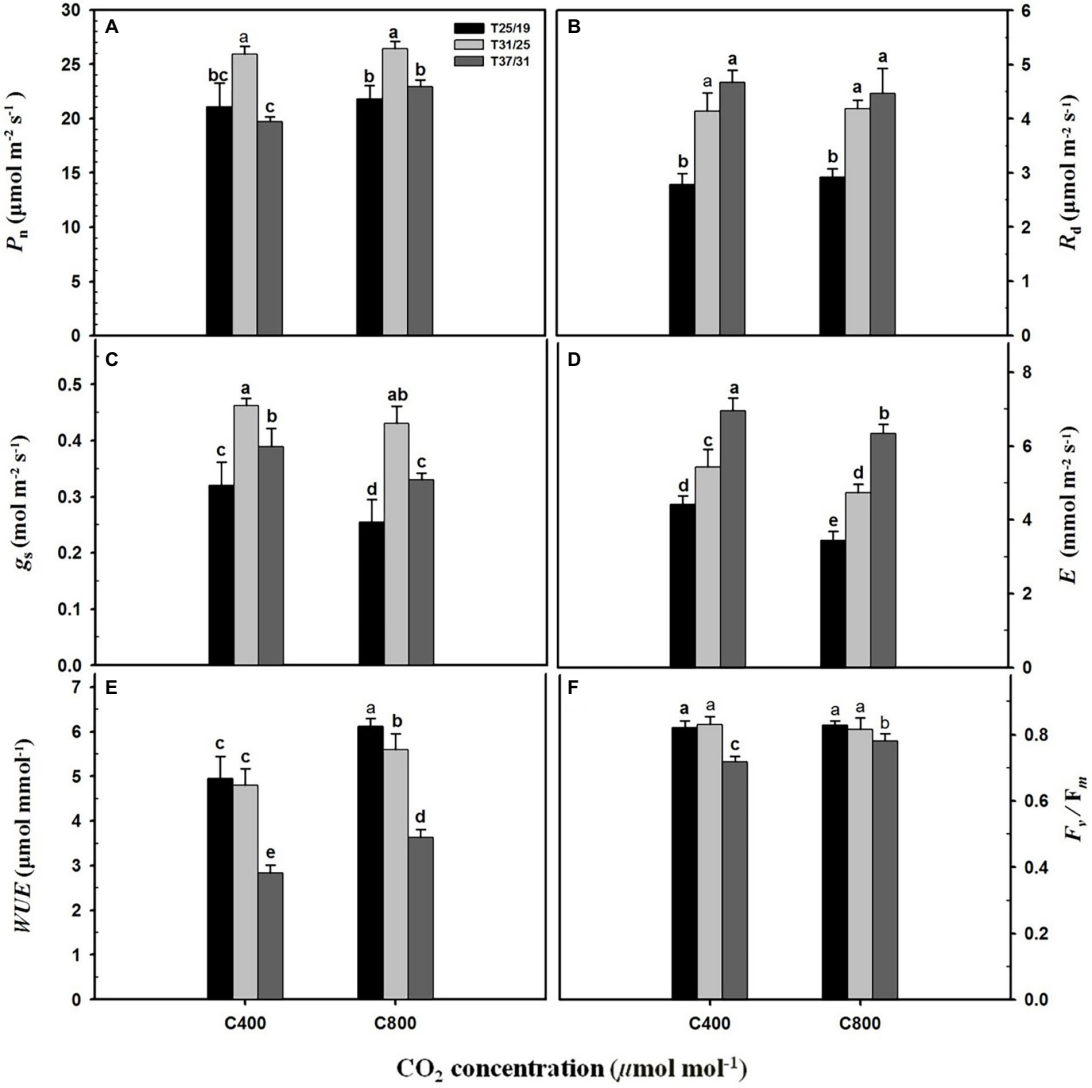
Leaf gas exchange parameters and chlorophyll fluorescence in response to the two CO_2_ concentrations and three temperature regimes. The different letters represent statistical differences at *p* < 0.05. **(A)** Leaf net photosynthetic rate (*P*_n_); **(B)** Leaf dark respiration rate (*R*_d_); **(C)** Stomatal conductance (*g*_s_); **(D)** Leaf transpiration rate (*E*); **(E)** Leaf water use efficiency (*WUE*); **(F)** *F*_v_/*F*_m_ (maximum photochemical efficiency of PS*II*).

Elevated CO_2_ concentration (*e*[CO_2_]) substantially enhanced the *P*_n_ rate by 16.4% under the highest temperature regime (37/31°C), whereas leaf photosynthesis of maize plants under the lower temperature regimes (31/25°C and 25/19°C) was not significantly affected by elevated CO_2_ concentration (Figure 2A). The *R*_d_ was substantially increased by 107 and 67% under *a*[CO_2_] and *e*[CO_2_] with increasing growth temperature from 25/19°C to 37/31°C (Figure 2B), but no significant difference was detected between *a*[CO_2_] and *e*[CO_2_] (Figure 2B). Elevated CO_2_ concentration decreased the *g*_s_ of maize leaves grown at the temperature regimes of 25/19°C and 37/31°C by 21.8% and 15.1%, respectively, whereas the *g*_s_ was not changed by *e*[CO_2_] when plants were subjected to 31/25°C regimes (Figure 2C). Similarly, *e*[CO_2_] significantly decreased *E* by 22.0%, 12.7%, and 8.9% when these maize plants were grown at 25/19°C, 31/25°C, and 37/31°C regimes, respectively (Figure 2D). By contrast, elevated temperature strongly increased *E* and reached the maximum values of 6.9 mmol m^−2^ s^−1^ and 6.3 mmol m^−2^ s^−1^ at *a*[CO_2_] and *e*[CO_2_] under 37/31°C regimes (Figure 2D). As a result, enriching CO_2_ concentration dramatically enhanced the *WUE* of maize plants by 24.5%, 16.5%, and 27.4% under 25/19°C, 31/25°C, and 37/31°C regimes, respectively (Figure 2E). The *F*_v_/*F*_m_ was significantly decreased by elevated temperatures with the minimal values of *F*_v_/*F*_m_ under the highest temperature regime (37/31°C) regardless of [CO_2_] (Figure 2F). However, the *F*_v_/*F*_m_ value of maize leaves under *e*[CO_2_] was 20% higher than that of plants under *a*[CO_2_], when these maize plants were exposed to the highest temperature regime of 37/31°C (Figure 2F).

### Effects of CO_2_ concentration and temperature on morphological traits of individual stoma

**Figure 3:**
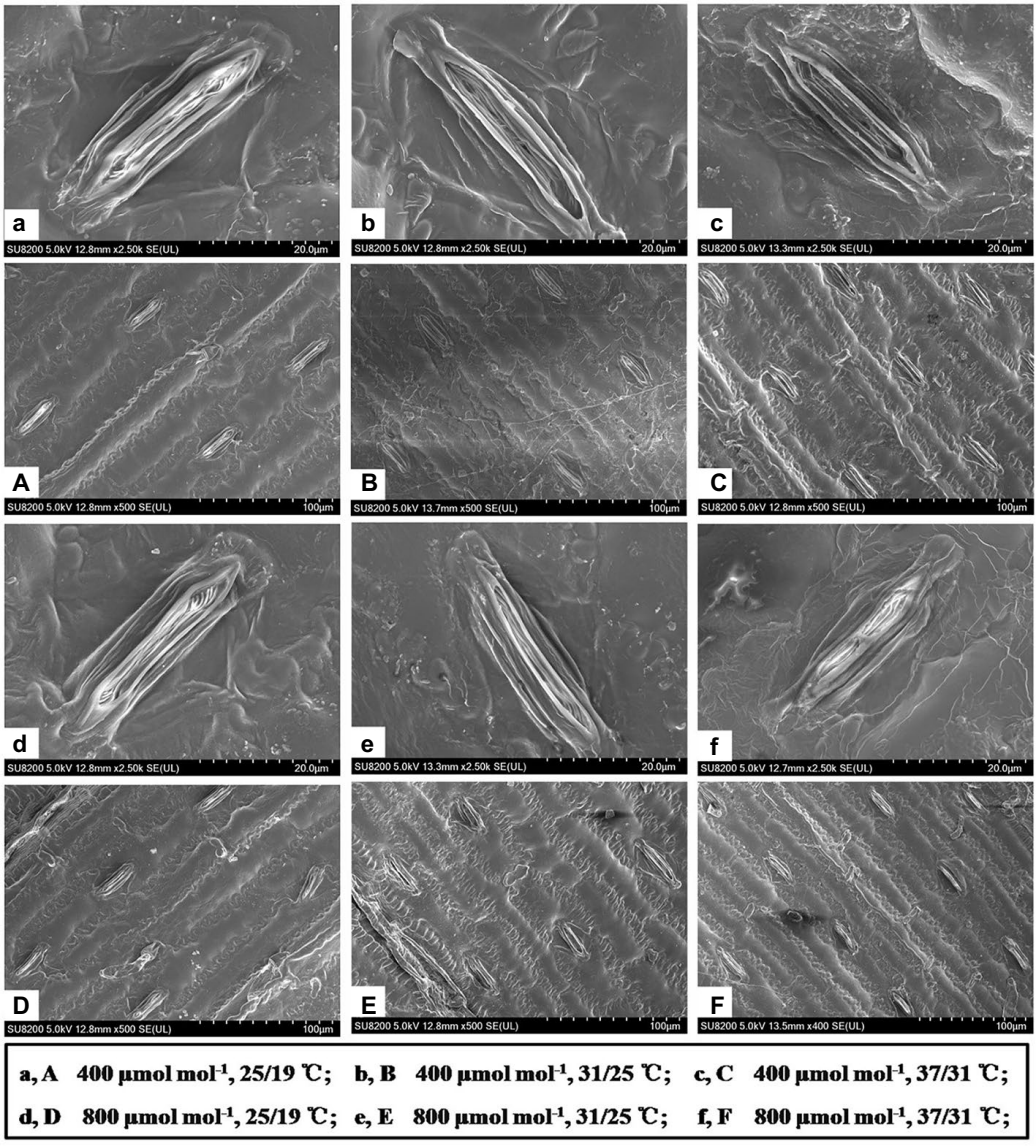
Scanning electron microscopy showed the stomatal characteristics of maize leaves grown at two CO_2_ concentrations and three temperatures regimes. Note that maize plants grown under 31/25°C and 37/31°C had much more smaller stomata than those of plants under 25/19°C. Bar = 20 μm **(a–f)** and Bar = 100 μm **(A–F)**.

The stomatal density was enhanced by the increase in growth temperatures (Table 2). Elevating the growth temperatures of maize plants from 25/19°C to 31/25°C and 37/31°C substantially increased the stomatal density by 13% and 40% under *a*[CO_2_] (Table 2). However, elevated CO_2_ concentration not significantly affected the stomatal density of maize plants under the three growth temperatures (Figure 3; Table 2). In contrast to stomatal density, elevated CO_2_ concentration substantially decreased stomatal area by 7% and 10% under the growth temperature regimes of 25/19°C and 37/31°C mainly due to the smaller stomatal width, although the stomatal area was barely altered by enriched CO_2_ concentration when maize plants were subjected to the 31/25°C regime (Table 2). Additionally, we also found a significant interactive effect of [CO_2_] and temperature on the stomatal width of maize plants (Table 2).

**Table 2:** Effects of elevated [CO_2_] and temperature on the morphological traits of individual stoma on maize leaves.

[CO_2_] (μ mol mol^−1^)	Temperature (°C)	Stomatal length (μm)^*^	Stomatal width (μm)^*^	Stomatal area (μm^2^)	Stomatal density (No. mm^−2^)	Stomatal area index (μm^2^ mm^−2^)^**^
	25/19	32.5 (2.3)^a^	4.8 (0.4)^a^	156 (17)^a^	87 (9)^c^	1.36 (0.22)^a^
400	31/25	31.5 (1.4)^ab^	4.6 (0.3)^b^	138 (13)^b^	98 (10)^b^	1.35 (0.16)^a^
	37/31	27.2 (1.1)^c^	4.1 (0.2)^c^	ww111 (8)^c^	122 (9)^a^	1.36 (0.16)^a^
	25/19	32.2 (1.5)^a^	4.6 (0.3)^b^	145 (13)^b^	88 (8)^c^	1.26 (0.11)^a^
800	31/25	29.9 (1.7)^b^	4.4 (0.3)^b^	138 (12)^b^	96 (9)^b^	1.33 (0.18)^a^
	37/31	27.3 (1.8)^c^	3.7 (0.3)^d^	100 (9)^d^	123 (9)^a^	1.23 (0.13)^a^
[CO_2_]		0.77	0.04	0.04	0.72	0.43
Temperature	*p*-values	0.001	0.001	0.001	0.001	0.04
*C* × *T*		0.81	0.04	0.075	0.99	0.27

Values given are means (standard deviation) for stomatal length (*n* = 48), stomatal width (*n* = 48), stomatal area (*n* = 48), stomatal density (*n* = 24), and stomatal area index (*n* = 24). Mean values were compared with Student–Newman–Keuls. Different letters indicate *p* < 0.05 and the same letters indicate *p* > 0.05.

* Stomatal length is the longest dimension, and the stomatal width is the widest dimension.

** Stomatal area index is defined as the total stomatal area per unit leaf area calculated as stomatal average density × stomatal average area per sample × 100%.

### Effects of CO_2_ concentration and temperature on leaf anatomy and chemical compositions

**Figure 4:**
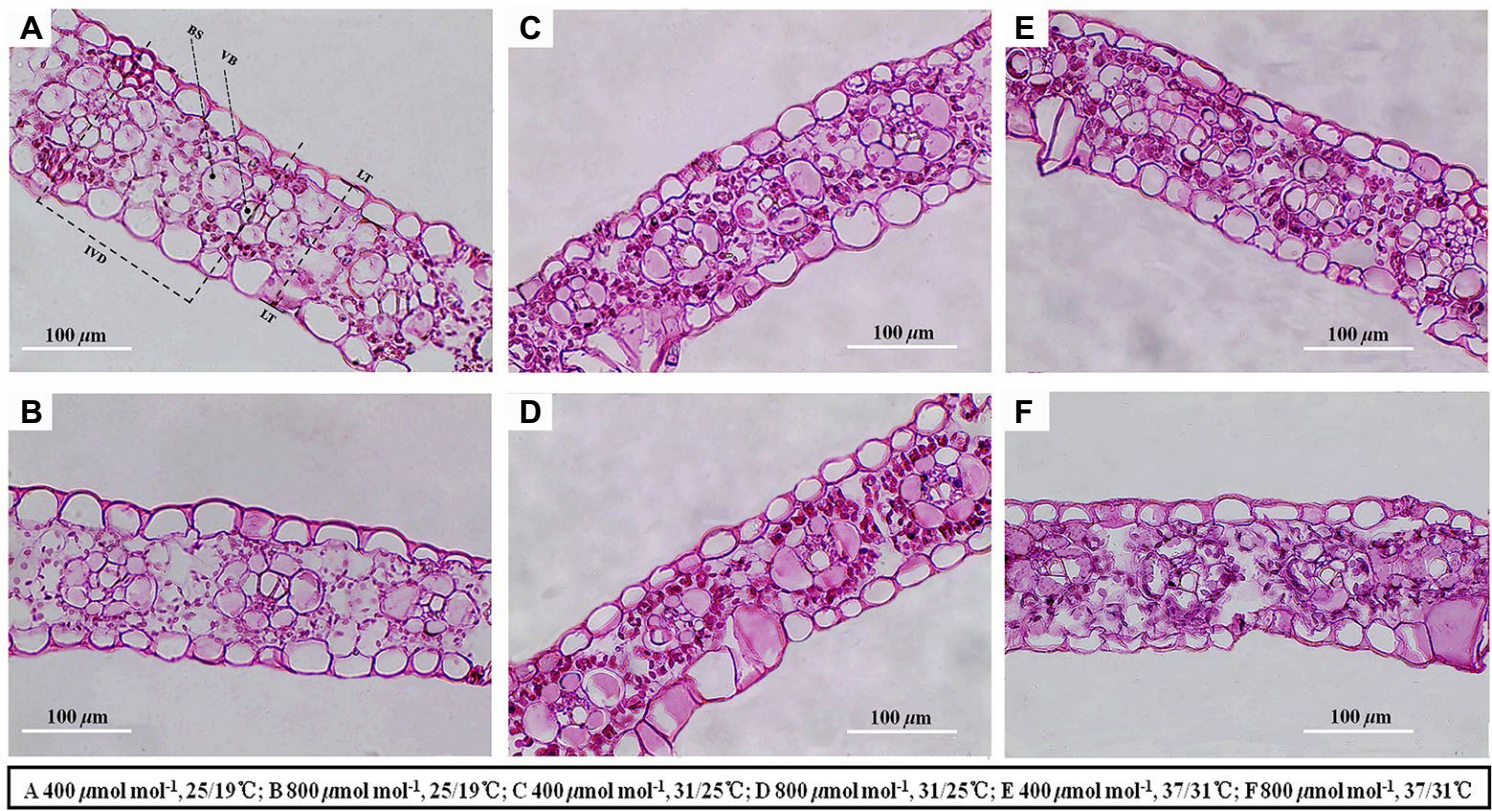
Light micrographs of cross sections showed the anatomical structure traits of maize leaves. Note that maize plants exposed to 25/19°C have greater leaf thickness, interveinal distance, bundle sheath tissue area and vascular bundle tissue area as compared with plants subjected to 31/25°C and 37/31°C. BS, bundle sheath; VB, vascular bundle; IVD, interveinal distance; LT, leaf thickness. Bar = 100 μm. The **(A–F)** represents different temperature and CO_2_ treatments.

Elevated temperature significantly decreased the leaf thickness, interveinal distance, vascular bundle tissue area and bundle sheath tissue area of maize plants, whereas vascular bundle index and bundle sheath index increased regardless of CO_2_ concentrations (Figure 4; Table 3). Meanwhile, elevated CO_2_ concentration significantly decreased the vascular bundle index of maize by 6.7% and 7.9% under 25/19°C and 37/31°C, respectively (Table 3). Moreover, elevated CO_2_ concentration had little effect on the leaf thickness, vascular bundle tissue area and bundle sheath tissue area under the three temperatures, whereas the interveinal distance of maize declined by 7.3% under 37/31°C (Figure 4; Table 3). Our two-way ANOVA results showed that *e*[CO_2_] substantially increased the interveinal distance and decreased vascular bundle index, while the leaf thickness, vascular bundle tissue area and bundle sheath tissue area showed little change (Table 3). Moreover, elevated temperature drastically decreased the leaf thickness, interveinal distance, vascular bundle tissue area, bundle sheath tissue area, vascular bundle index, and bundle sheath index (Table 3).

**Table 3:** Effects of elevated [CO_2_] and temperature on the anatomical structure of maize leaves.

[CO_2_] (μ mol mol^−1^)	Temperature (°C)	Leaf thickness (μm)	Interveinal distance (μm)	Vascular bundle tissue area (μm^2^)	Vascular bundle index^*^	Bundle sheath tissue area (μm^2^)	Bundle sheath index^**^
	25/19	140 (6)^a^	150 (8)^a^	1,260 (72)^a^	6.0 (0.5)^c^	4,042 (319)^a^	19.3 (1.8)^c^
400	31/25	131 (6)^bc^	123 (8)^b^	1,150 (93)^b^	7.2 (0.6)^b^	3,701 (239)^b^	23.1 (2.4)^b^
	37/31	127 (5)^c^	110 (5)^c^	1,058 (67)^c^	7.6 (0.5)^a^	3,570 (287)^b^	25.7 (2.6)^a^
	25/19	143 (6)^a^	153 (7)^a^	1,224 (85)^a^	5.6 (0.3)^d^	4,147 (331)^a^	19.1 (1.7)^c^
800	31/25	133 (6)^b^	124 (9)^b^	1,118 (79)^bc^	6.8 (0.6)^b^	3,805 (206)^b^	23.3 (2.6)^b^
	37/31	129 (6)^bc^	118 (8)^b^	1,052 (81)^c^	7.0 (0.5)^b^	3,615 (208)^b^	24.0 (2.4)^b^
[CO_2_]		0.16	0.03	0.12	0.001	0.13	0.21
Temperature	*p*-values	0.001	0.001	0.001	0.001	0.001	0.001
*C* × *T*		0.96	0.17	0.71	0.41	0.88	0.22

Values given are means (standard deviation) for leaf thickness (4 leaves × 2 images/leaf × 2 thicknesses/image = 16 thicknesses; *n* = 16), interveinal distance (4 leaves ×2 images/leaf × 2 interveinal distances/image = 16 interveinal distances; *n* = 16), vascular bundle tissue area (4 leaves ×2 images/leaf × 2 vascular bundle tissue areas/image = 16 vascular bundle tissue areas; *n* = 16), vascular bundle index (4 leaves ×2 images/leaf × 1 vascular bundle index/image = 8; *n* = 8), bundle sheath tissue area (4 leaves ×2 images/leaf × 2 bundle sheath tissue areas/image = 16; *n* = 16), and bundle sheath index (4 leaves ×2 images/leaf × 1 bundle sheath index/image = 8; *n* = 8). Mean values were compared with Student–Newman–Keuls. Different lowercase letters indicate *p* < 0.05 and the same lowercase letters indicate *p* > 0.05.

* Vascular bundle index was defined as the vascular bundle tissue area per unit leaf cross-sectional area calculated as average vascular bundle tissue area/ (interveinal distance × leaf thickness) × 100%.

** Bundle sheath index was defined as the bundle sheath tissue area per unit leaf cross-sectional area calculated as average bundle sheath tissue area/ (interveinal distance × leaf thickness) × 100%.

Elevated CO_2_ concentration drastically enhanced the total soluble sugar concentration by 23.2% under 37/31°C regime, due mainly to the increased concentrations of fructose, sucrose, and glucose (Table 4), whereas the total soluble sugar content was not significantly affected by elevated CO_2_ concentration under the temperature regimes of 25/19°C and 31/25°C (Table 4). Nevertheless, increasing the growth temperature from 25/19°C to 37/31°C substantially decreased the total soluble sugar concentration by 28% and 20% under *a*[CO_2_] and *e*[CO_2_], respectively. Furthermore, elevated CO_2_ concentration had little impact on the starch concentration in maize leaves under the three temperature regimes. By contrast, elevated temperature significantly decreased all the documented chemical composition parameters in maize leaves. In addition, the concentrations of glucose, fructose and the nonstructural carbohydrates were statistically different between the two CO_2_ concentrations (Table 4).

**Table 4:** Effects of elevated [CO_2_] and temperature on leaf chemical compositions of maize plants.

[CO_2_] (μ mol mol^−1^)	Temperature (°C)	Soluble Sugars (%, w/w)				Starch (%, w/w)	Non-structural carbohydrates (%, w/w)
		Fructose	Sucrose	Glucose	Total soluble sugars		
	25/19	0.93 (0.09)^b^	0.76 (0.08)^bc^	1.42 (0.12)^a^	3.11 (0.22)^b^	3.61 (0.12)^a^	6.72 (0.26)^a^
400	31/25	1.14 (0.10)^a^	0.86 (0.04)^a^	1.52 (0.18)^a^	3.51 (0.23)^a^	3.21 (0.13)^bc^	6.73 (0.13)^a^
	37/31	0.69 (0.06)^c^	0.52 (0.05)^d^	1.02 (0.13)^c^	2.24 (0.22)^d^	3.13 (0.18)^bc^	5.36 (0.32)^b^
	25/19	1.24 (0.08)^a^	0.75 (0.05)^bc^	1.46 (0.09)^a^	3.45 (0.17)^ab^	3.68 (0.11)^a^	7.13 (0.24)^a^
800	31/25	1.23 (0.04)^a^	0.83 (0.04)^ab^	1.64 (0.15)^a^	3.70 (0.20)^a^	3.29 (0.25)^b^	6.99 (0.39)^a^
	37/31	0.87 (0.06)^b^	0.67 (0.04)^c^	1.22 (0.08)^b^	2.76 (0.17)^c^	2.97 (0.11)^c^	5.73 (0.27)^b^
[CO_2_]		0.001	0.13	0.03	0.01	0.96	0.01
Temperature	*p*-values	0.001	0.001	0.001	0.001	0.001	0.001
*C* × *T*		0.11	0.07	0.48	0.35	0.25	0.89

Values are means (standard deviation) for fructose (4 plants × 1 leaf/plant = 4 leaves; *n* = 4), sucrose (4 plants × 1 leaf/plant = 4 leaves; *n* = 4), glucose (4 plants × 1 leaf/plant = 4 leaves; *n* = 4), total soluble sugars (4 plants × 1 leaf/plant = 4 leaves; *n* = 4), starch (4 plants × 1 leaf/plant = 4 leaves; *n* = 4), and non-structural carbohydrates (4 plants × 1 leaf/plant = 4 leaves; *n* = 4). Mean values were compared with Student–Newman–Keuls. Different lowercase letters indicate *p* < 0.05 and the same lowercase letters indicate *p* > 0.05.

### Relationships among leaf photosynthesis, stomatal characteristics, leaf transpiration rate and leaf anatomic structure

**Figure 5:**
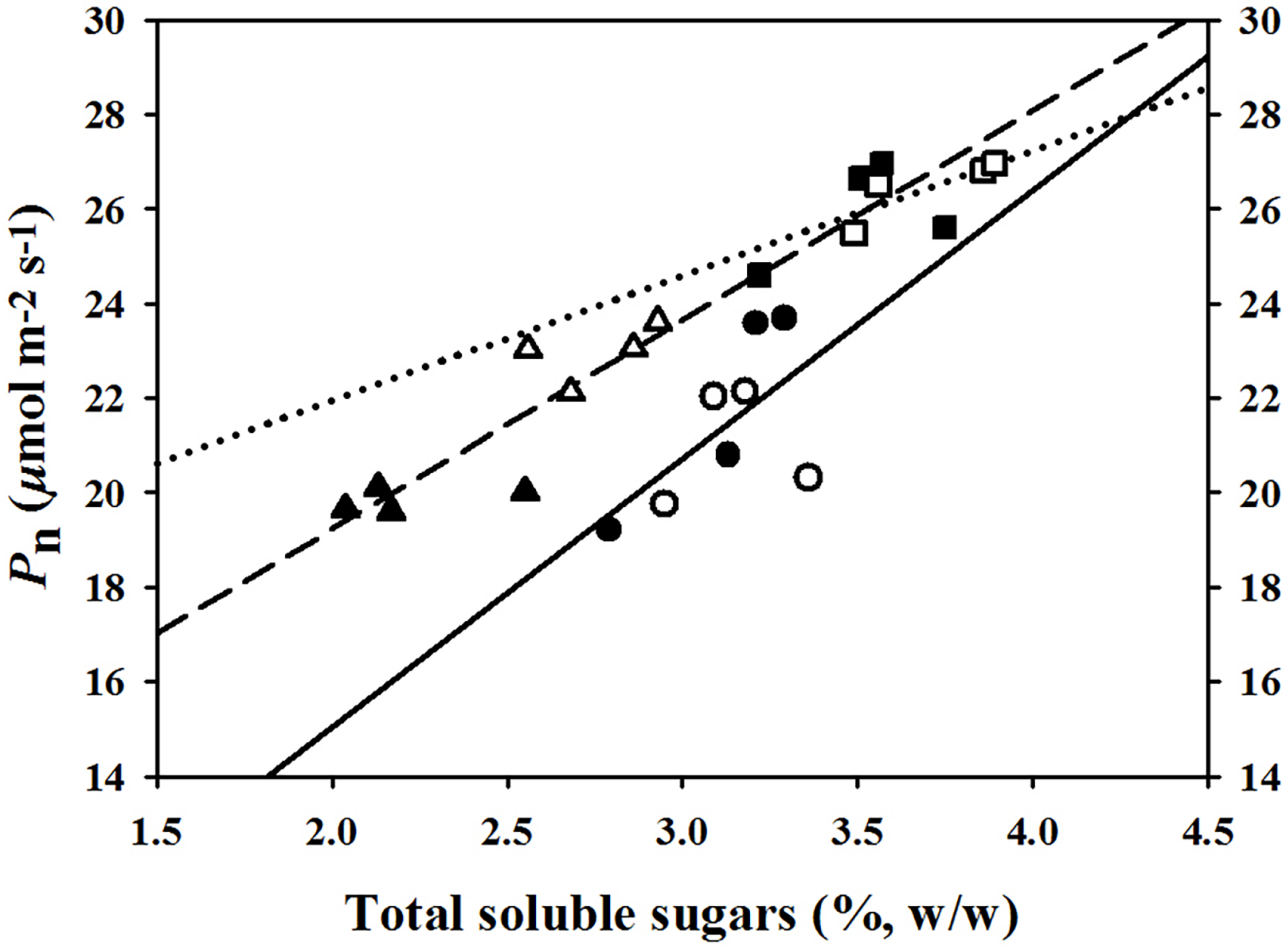
The fitted relationships between net photosynthetic rates (*P*_n_) and total soluble sugar contents under the temperature regimes of 25/19°C, 31/25°C, and 37/31°C. The solid line and circles, dotted line and squares as well as medium dash line and triangles represent the fitted functions under the temperature regimes of 25/19°C (*R*^2^ = 0.38), 31/25°C (*R*^2^ = 0.45), and 37/31°C (*R*^2^ = 0.77), respectively. The closed and open symbols (circles, triangles, and squares) indicate these fitted points from ambient [CO_2_] or elevated [CO_2_].

**Figure 6:**
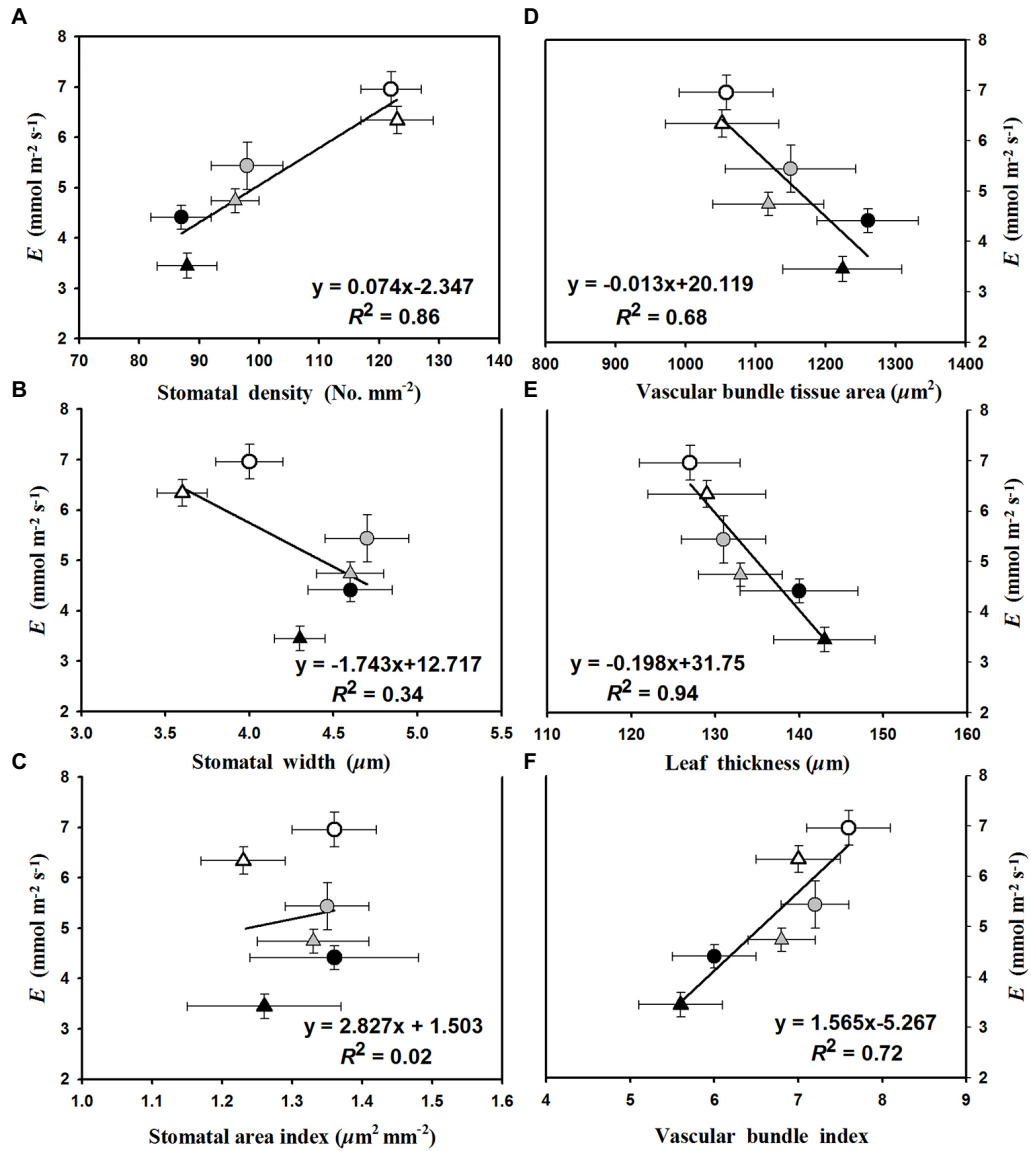
The fitted relationships of leaf transpiration rates (*E*) with **(A)** stomatal density, **(B)** stomatal width, **(C)** stomatal area index, **(D)** vascular bundle tissue area, **(E)** leaf thickness, and **(F)** vascular bundle index. The circles and triangles represent the data from ambient or elevated [CO_2_] and the black, gray, and white symbols (circles and triangles) indicate the fitted points under the temperature regimes of 25/19°C, 31/25°C, and 37/31°C.

The linear regression relationships between leaf photosynthesis and total soluble sugars were estimated under the three temperature regimes of 25/19°C, 31/25°C, and 37/31°C (Figure 5). We found linearly positive relationships between *P*_n_ and total soluble sugars with *R*^2^ values of 0.38, 0.45, and 0.77 at the temperature regimes of 25/19°C, 31/25°C, and 37/31°C, respectively (Figure 5). The *E* was linearly enhanced by the increases of stomatal density (Figure 6A) and vascular bundle index (Figure 6F), whereas a negative linear relationship was obtained among *E* and stomatal width (Figure 6B), vascular bundle tissue area (Figure 6D) as well as leaf thickness (Figure 6E). However, the stomatal area index was not linearly related with the transpiration rates of maize plants (Figure 6C).

## Discussion

### Effects of elevated temperature on leaf photosynthesis and transpiration of maize plants

Most plant species have an optimal growth temperature and only grow in a certain temperature range (Jin et al., 2011). Therefore, temperature above the optimum may result in leaf photosynthetic decline (Hao et al., 2019) through damaging the structure of the chloroplast and photosystem II (Janka et al., 2015) and suppressing the active state of Rubisco (Yamori et al., 2012). Our results that *P*_n_ was significantly enhanced with increasing temperature from 25/19°C to 31/25°C, and then dramatically declined when the temperature was continually increased to 37/31°C, indicated that the optimal temperature for leaf photosynthesis of maize plants is around 31/25°C regime. Interestingly, Kim et al. (2007) also estimated the optimal temperature for leaf photosynthesis and plant growth of maize is about 34°C. Therefore, these results also suggested that maize plants might suffer high temperature stress with increasing the temperature from 31/25°C to 37/31°C. The *F*_v_/*F*_m_ values under 37/31°C were the lowest among the three temperature regimes which also directly supported the above conclusions that *P*_n_ was inhibited when maize plants were exposed at the highest temperature regime of 37/31°C. Furthermore, the *E* of maize was substantially increased by elevating growth temperature from 25/19°C to 37/31°C under both CO_2_ concentrations, suggested that maize plants may adapt to high temperatures through enhancing leaf transpiration at the same [CO_2_] levels, because higher transpiration rates can take much more heat from leaf water loss, and thus protect the reaction site of leaf photosynthesis from high temperature stress.

### Elevated [CO_2_] enhanced leaf photosynthesis and water use efficiency of maize plants with changes in leaf anatomy and stomatal traits under high temperatures

Several studies have found that the leaf photosynthesis of several C_4_ species responds positively to CO_2_ enrichment (Leakey et al., 2004; Driscoll et al., 2005; Abebe et al., 2016), suggesting that the CO_2_ saturation point of leaf photosynthesis for C_4_ species might be dependent on a number of environmental conditions such as growth temperatures (Sage, 2002), soil water availability (Kellner et al., 2019) and vapor pressure deficit (Maherali et al., 2010). Our results also showed that elevated CO_2_ concentration significantly increased the *P*_n_ of maize under high temperature regime (37/31°C), whereas CO_2_ enrichment had little effect on the leaf photosynthesis of maize plants grown at the low and moderate temperature regimes (25/19°C and 31/25°C; Figure 2A), indicating that leaf-level photosynthesis of maize plants in response to elevated CO_2_ concentration was highly temperature-dependent. These findings were consistent with previous conclusions on *Amaranthus* (Sage, 2002), where the CO_2_ saturation point was increased with the elevated growth temperature, and thus the sensitivity of leaf photosynthesis to CO_2_ enrichment in C_4_ plants might be enhanced by elevated temperatures (Sage, 2002). Also, the increased *P*_n_ of maize plants under elevated CO_2_ concentrations may be attributed to the increased CO_2_ saturation point under the high temperature regime in the current study. However, it should be noted that the activity and capacity of Rubisco for C_4_ plants are also closely related to their growth temperatures (Perdomo et al., 2017).

We found that elevated temperature reduced the leaf thickness and interveinal distance in maize leaves, which may benefit maize plants by enhancing leaf transpiration rates under high temperatures as observed in the current study, because thinner leaves may allow for easier transfer of water from leaf surface to atmosphere, and meanwhile shorter interveinal distance is likely to increase the number of vascular bundles in leaves and thus enhance the efficiency of H_2_O diffusion. In addition, we also found that elevated temperature enhanced leaf transpiration of maize leaves is highly dependent on the increase in stomatal density of maize plants, because having more stomata reduces the distance between mesophyll cells and stomata, thus decreasing the resistance of mesophyll tissues to H_2_O diffusion (Zheng et al., 2013). Finally, the changes in stomata and leaf structures resulted in the increase in *E* with elevated temperature from 25/19°C to 37/31°C at both the *a*[CO_2_] and *e*[CO_2_], and thus reduced the leaf water use efficiency of maize plants. Furthermore, we found a positive relationship between stomatal density and leaf transpiration, although leaf thickness was negatively related to leaf transpiration. Similarly, we also found a positive relationship between vascular bundle index and transpiration rates, which indirectly supported the above conclusion that the declines of leaf transpiration rates may be attributed to the reduced vascular bundle index under higher CO_2_ concentration. In addition, our results that elevated temperature increased stomatal density and decreased leaf thickness suggested that maize plants respond to elevated temperature by enhancing leaf transpiration through changes in leaf anatomy and stomatal traits.

In the current study, our results showed that the vascular bundle index was significantly reduced by elevated CO_2_ concentration, which might partially contribute to the decline in leaf transpiration under higher CO_2_ concentration, because vascular bundles are closely related to water transport efficiency (Yang and Liang, 2008). Interestingly, our results that elevated CO_2_ concentration also reduced stomatal width and stomatal area of maize plants under the highest temperature regime of 37/31°C, indicated that the smaller stomatal openness may also contribute to the reduction in leaf transpiration, because maize plants can benefit from smaller stomata through maintaining tissue turgor (Rodriguez-Dominguez et al., 2016) and leaf water potential (Kumar et al., 2005) of maize under higher temperatures. This decreased leaf transpiration may also partially contribute to the enhanced water use efficiency under high CO_2_ concentration (Hussain et al., 2013), although leaf photosynthesis was substantially increased by elevated CO_2_ concentration under high temperatures.

### Changes in foliar nonstructural carbohydrates explain the CO_2_ fertilization effect on maize plants under high temperature conditions

It has been widely evident that the physiological and biochemical responses of plants to environmental factors such as temperature and CO_2_ concentration are highly related to the carbon source-sink balance (Inman-Bamber et al., 2010; Sugiura et al., 2017), where the changes of carbohydrates may have profound effects on leaf photosynthesis through various processes including both up and downregulations (Nebauer et al., 2011; Zheng et al., 2013; Sugiura et al., 2017). Elevating the growth temperature from 25/19°C to 31/25°C significantly increased the soluble sugars in maize leaves mainly due to the enhanced *P*_n_ of maize plants. However, the leaf soluble sugars were drastically decreased with further elevating growth temperature from 31/25°C to 37/31°C, indicating that high temperature stress might inhibit leaf photosynthesis as demonstrated by the declines in the soluble sugar of maize leaves.

Response of leaf photosynthesis to elevated CO_2_ concentration is also highly dependent on the source-sink balance of carbohydrates, because the excessive carbohydrate accumulation can reduce both the efficiency and content of Rubisco through sucrose cycling and photosynthetic gene repression of plants under higher CO_2_ concentrations (Córdoba et al., 2017), and thus the imbalance between sink and source carbohydrates may lower the CO_2_ fertilization effect on leaf photosynthesis (Fan et al., 2020). Elevated CO_2_ concentration can drastically enhance the growth and crop yield of plants through the “CO_2_ fertilization effect,” which is attributed to the increases of starch and soluble sugars in plant leaves (Pérez-Jiménez et al., 2017; Habermann et al., 2019). Interestingly, our results showed that the soluble sugars in maize leaves were substantially increased by elevated CO_2_ concentration even at the highest temperature regime of 37/31°C. These results suggested that high temperature may constrain the activation status of enzymes such as cellulose synthase (Suseela et al., 2014), which can transfer nonstructural carbohydrates into woody tissues (lignin and cellulose; Chen et al., 2012), because the leaf carbohydrates of maize plants were significantly increased by elevated CO_2_ concentration, but the total biomass was not statistically different between the two CO_2_ concentrations at all of the three growth temperature regimes in the current study. The fact that maize plants stored much more soluble sugars under high temperature may also be an adaptive strategy for surviving high temperature stress, because the storage of carbohydrates in plant leaves can reduce the heat sensitivity of photosynthetic electron transport (Hüve et al., 2010) and meanwhile protect photosynthetic organs such as chloroplasts from high temperature stress (Huang et al., 2015). Overall, our results that maize plants under high temperature stress had higher leaf photosynthesis and *F*_v_/*F*_m_ values at elevated CO_2_ concentration than those of plants at ambient CO_2_ concentration indicated that elevated CO_2_ concentration can partially alleviate the high temperature stress on maize plants through increasing the carbohydrates in maize leaves (Hüve et al., 2010). This conclusion could be supported by the positive relationships between leaf photosynthesis and soluble sugars under the highest temperature regime of 37/31°C.

The CO_2_ fertilization effect on the growth and grain yield of maize may also be confounded with other environmental factors, such as water availability (Blango et al., 2019), nitrogen deposition (Churkina et al., 2009), and ozone concentration (Cao et al., 2009), which may lower or even offset the CO_2_ fertilization effect on global agriculture productivity under future climate change. Unfortunately, this confounding effect is already happening and will gradually become worse under future climate change, because elevated atmospheric CO_2_ concentration and temperature may come with increasing nitrogen deposition (Churkina et al., 2009) and regional drought (Contran et al., 2012) in most temperate regions throughout the world where maize grows. Meanwhile, it should be noted that the responses of maize plants to the elevated CO_2_ concentration and temperature in the real world may be different from the findings of our study (Ainsworth et al., 2008; Poorter et al., 2016). In addition, this study mainly focused on the interactive effects of elevated CO_2_ concentration and temperature on the vegetative growth of maize plants, but we did not continue the experiment to further examine the impacts of elevated CO_2_ concentration and temperature on grain yield due to the height limitation of our growth chambers and the physiological stresses on maize grown in high temperatures. Nevertheless, the vegetative growth of maize plants is the most important foundation for yield production, thus the vegetative growth of maize in response to elevated CO_2_ concentration and temperature can be used to predict the potential impacts of future climate change on the grain yield of maize (Kim et al., 2007). Therefore, further studies with long-term controlled experiments in natural conditions are needed to fully understand the potential mechanisms and processes governing the interactive effects of elevated CO_2_ concentration and temperature on maize plants for improving the predictions of future climate change on maize production.

It should be noted that this study focused primarily on the effects of elevated CO_2_ concentration and temperature on the morphology, physiology, and biochemistry of maize plants, and we did not continue the experiment to further evaluate the genomic and/or proteomic responses of maize plants to elevated CO_2_ concentration and temperature. Therefore, based on the morphological, physiological, and biochemistrical results and conclusions from the current study, further studies with genomic and proteomic approaches are needed to fully understand the underlying mechanisms and processes of combined impacts of elevated CO_2_ concentration and temperature on maize plants under future climate change. Additionally, it is noted that this study is a pot-based manipulation experiment with environmental growth chambers, where the four pots in each environmental growth chamber were treated as four replicates, and thus the significant differences among different treatments might potentially be affected by “pseudo-replication.” Nevertheless, we adopted a widely used method to minimize this spatial “pseudo-replication” effects from different environmental growth chambers through randomly changing the CO_2_ concentration and temperature of each growth chamber, and meanwhile relocating the treated maize plants to the environmental growth chambers with corresponding CO_2_ concentrations and temperatures every week during the treatment period in the current study (Xu et al., 2009; Jin and Evans, 2010; Yu et al., 2012, 2017).

## Conclusion

We found a very strong temperature effect on biomass accumulation of maize plants with increasing plant biomass at lower temperatures below their optimal temperature and decreasing plant biomass when the temperature is beyond the optimum. Meanwhile, we also found CO_2_ fertilization effects on plant growth and leaf photosynthesis of maize under the highest temperature regime (37/31°C), but this CO_2_ fertilization effect diminished under the two lower growth temperature regimes. These results indicate that the CO_2_ fertilization effect on plant growth and leaf photosynthesis of maize depended on growth temperatures, and the high temperature stress on maize plants could also be partially alleviated by elevated CO_2_ concentration through changing stomatal traits, leaf anatomy, and nonstructural carbohydrates. Overall, our results suggest that maize plants may suffer less from future high temperature stress by taking advantage of the CO_2_ fertilization effect. Nevertheless, it is noted that our findings were from a controlled environmental growth chamber experiment with only one maize cultivar., and thus further field-based experiments with many more maize cultivars under strongly varying conditions are needed to fully understand the potential mechanisms and processes of maize plants in response to the combined effects of elevated CO_2_ concentration and temperature.

## Data availability statement

The original contributions presented in the study are included in the article/supplementary material, further inquiries can be directed to the corresponding author.

## Author contributions

LL, LH, and YZe designed the study. LL, LH, YZa, HZ, BM, YT, and ZC performed the experiment. LL, LH, YT, YZa, YC, and YZe analyzed the data. LL, BM, HZ, and YZe wrote the initial manuscript. All authors contributed to the article and approved the submitted version.

## Funding

This research was partially supported by the National Natural Science Foundation of China (grant nos. 32071608 and U1802241), the Natural Science Foundation of Hebei Province (E2021402031), and the Central Guidance on Local Science and Technology Development Funding of Hebei Province (226Z6401G).

## Conflict of interest

The authors declare that the research was conducted in the absence of any commercial or financial relationships that could be construed as a potential conflict of interest.

## Publisher’s note

All claims expressed in this article are solely those of the authors and do not necessarily represent those of their affiliated organizations, or those of the publisher, the editors and the reviewers. Any product that may be evaluated in this article, or claim that may be made by its manufacturer, is not guaranteed or endorsed by the publisher.
